# Peri-Procedural Continuation Versus Interruption of Anticoagulation for Transcatheter Aortic Valve Implantation: A Systematic Review and Meta-Analysis

**DOI:** 10.3390/jcm14103563

**Published:** 2025-05-20

**Authors:** Jacinthe Khater, Marco Frazzetto, Filippo Luca Gurgoglione, Jasim Hasan, Davide Donelli, Guilherme Attizzani, Bernardo Cortese

**Affiliations:** 1 DCB Academy, 20143 Milan, Italy; jacinthekhater1234@hotmail.com (J.K.); marcofrazzetto7@gmail.com (M.F.); filippolucagurgoglione@gmail.com (F.L.G.); j.langawi@gmail.com (J.H.); 2 Faculty of Medical Sciences, Rafic Hariri University Campus, Lebanese University, Hadath 6573, Lebanon; 3 Department of Cardiology, University Hospitals Cleveland Medical Center, Cleveland, OH 44106, USA; guilherme.attizzani@uhhospitals.org; 4 Division of Cardiology, Parma University Hospital, University of Parma, 43126 Parma, Italy; donelli.davide@gmail.com; 5 CHU Amiens-Picardie, 80000 Amiens, France; 6 Mohammed Bin Khalifa Bin Salman Al Khalifa Specialist Cardiac Centre (MKCC), Awali P.O. Box 101, Bahrain

**Keywords:** periprocedural anticoagulation, TAVI, continuation vs. interruption, atrial fibrillation, bleeding outcomes

## Abstract

**Background/Objectives**: Oral anticoagulation therapy (OAC) is crucial for reducing the risk of ischemic complications in patients with atrial fibrillation (AF). However, OAC also increases the risk of major bleeding events. The optimal management of OAC in patients with AF undergoing transaortic valve implantation (TAVI) is unclear. This study aimed to compare the efficacy and safety of OAC interruption vs. continuation in patients with AF scheduled for TAVI. **Methods**: PubMed, EMBASE, and Cochrane were searched to include all pertinent randomized and observational studies. The primary endpoint was the occurrence of net adverse clinical events (NACE), a composite of all-cause death, major vascular complications, and major bleeding at 30-day follow-up. Secondary endpoints included all-cause death, cardiovascular death, major vascular complications, major bleeding, any bleeding, stroke, non-fatal myocardial infarction, and the need for red-packed blood transfusion. **Results**: A total of three studies and 2773 patients were included in the analysis (1314 were allocated to continuation of OAC therapy and 1459 to interruption of OAC therapy during TAVI). The two study groups experienced a similar rate of NACE (OR = 0.89 [95% CI 0.61 to 1.31], I^2^ = 77%, *p* = 0.56) compared to the OAC-interruption group. No significant differences were observed in the rate of all-cause death (*p* = 0.21), cardiovascular death (*p* = 0.35), major vascular complications (*p* = 0.84), major bleeding events (*p* = 0.47), total bleeding events (*p* = 0.62), or non-fatal MI (*p* = 0.55). Interestingly, the OAC-continuation group experienced a lower occurrence of stroke (OR = 0.62 [95% CI 0.39 to 0.97], I^2^ = 0%, *p* = 0.04) and the need for red packed blood cells (OR = 0.66 [95% CI 0.50 to 0.86], I^2^ = 20%, *p* < 0.01) compared to the OAC-interruption group. **Conclusions**: In patients with AF undergoing TAVI, there was no significant difference between interruption and continuation of OAC in terms of NACE, composite of all-cause death, major vascular complications, or major bleeding at 30-day follow-up. Of interest, the OAC-continuation group patients experienced lower rates of stroke and the need for blood transfusion.

## 1. Introduction

Transcatheter aortic valve implantation (TAVI) is an established therapy for patients with symptomatic severe aortic stenosis [1]. It constitutes the standard of care for older patients (≥75 years) and those who are at high risk or unsuitable for surgery.

Approximately one-third of patients scheduled for TAVI are treated with oral anticoagulation (OAC) therapy due to concomitant conditions, especially atrial fibrillation (AF) [2]. OAC therapy is crucial for reducing the risk of ischemic complications in this population; however, it also increases the risk of major bleeding events [3]. Furthermore, the TAVI procedure itself is associated with a significant risk of ischemic complications, mainly due to debris embolization [4,5], as well as major bleeding events, both at the access site and non-access locations [6,7].

With the growing number of TAVI procedures in OAC patients, determining the optimal management of OAC therapy during the periprocedural phase has become a key concern [8], requiring a delicate balance between ischemic and hemorrhagic risks. However, current guidelines remain elusive [2,9], and clinical evidence reports conflicting findings [10,11,12].

This systematic review and meta-analysis aimed to synthesize the current evidence on the continuation versus interruption of OACs during the periprocedural phase in patients who are being treated with OAC undergoing TAVI.

## 2. Materials and Methods

### 2.1. Protocol and Registration

This comprehensive review was conducted following the Cochrane Handbook for Systematic Reviews of Interventions and was reported according to the Preferred Reporting Items for Systematic Reviews Incorporating Network Meta-analyses (PRISMA-NMA) guidelines. The meta-analysis protocol was registered on PROSPERO (ID: CRD42024618742).

### 2.2. Eligibility Criteria

Inclusion in this meta-analysis was restricted to studies that met all the following eligibility criteria: (1) randomized trials or observational studies including patients scheduled for TAVI; (2) studies involving patients prescribed OACs (both direct oral anticoagulants (DOACs) and vitamin K antagonists (VKAs)) for concomitant comorbidities; (3) studies comparing interruption to continuation of OACs during the periprocedural phase of TAVI; (4) studies reporting clinical outcomes during the periprocedural phase and at 30-day follow-up.

### 2.3. Search Strategy and Data Extraction

The initial search included the Cochrane Library, EMBASE and PubMed from inception to November 2024 using the keywords (“Transcatheter Aortic Valve Implantation” OR “TAVI” OR “TAVR”) AND (“Continued Anticoagulation” OR “Anticoagulant Continuation”) AND (“Interrupted Anticoagulation” OR “Anticoagulant Interruption”) AND (“Thromboembolism” OR “Thromboembolic Events” OR “Bleeding Complications” OR “Procedure Success”) AND (“Vitamin K Antagonist” OR “VKA” OR “Warfarin” OR “Direct Oral Anticoagulants” OR “DOACs” OR “Factor Xa Inhibitors” OR “Dabigatran” OR “Rivaroxaban” OR “Apixaban” OR “Edoxaban”). The retrieval strategy was applied with customization of the search strings to accommodate the recommendations of each database. No document type or other relevant restrictions were used in the retrieval process, and unpublished articles were excluded. In addition, presentations from major cardiovascular meetings and references of the included studies were also screened. No search limitations by publication date, publication status, or language were applied. All the search results were imported into the Rayyan.ai software for the management of the review process. The screening process was conducted by two independent reviewers (JK and MF) who assessed titles and abstracts for relevance, followed by a full-text review to determine eligibility. A double-blind approach was used to minimize potential selection bias. Reviewer discrepancies were resolved through discussion or consultation with a third reviewer (BC). Only studies that included: (1) Adults ≥18 years; (2) Undergoing TAVI; (3) Indication of anticoagulation for long-term treatment of AF, patients with a mechanical valve or patients with DVT/PE or other rare causes; (4) studies with 2 arms showing a comparison of peri-procedural continuation vs. interruption of OACs; (5) Continuation defined as uninterrupted or minimally interrupted without bridging; (6) Reported outcomes: bleeding, stroke, mortality, valve thrombosis, or vascular complications; (7) RCTs or observational studies; (8) English language; (9) Full text available and available results. Furthermore, studies meeting any of the following exclusion criteria were excluded: (1) No direct comparison of continuation vs. interruption; (2) Non-TAVI procedures (e.g., SAVR); (3) Outcomes reported post-TAVI (beyond the 30 days limit period); (4) Case reports, reviews, editorials, or abstracts; (5) Animal or in vitro studies; (6) Bridging strategies only without continuation/interruption comparison; (7) Duplicate or overlapping data.

### 2.4. Quality Assessment

We evaluated the risk of bias in randomized and observational studies using version 2 of the Cochrane Risk of Bias assessment tool [13]. Disagreements were resolved through a consensus after discussing the reasons for the discrepancy.

### 2.5. Statistical Analysis

A quantitative analysis, consisting of three meta-analyses, was conducted using the software “R” (RStudio ver. 2022.07.2) and the package “meta”. Odds ratios (ORs) and confidence intervals (CIs) were employed as a measure of effect size. The threshold for statistical significance of the overall effect size was set at *p* < 0.05. A random-effects model was employed, and the DerSimonian and Laird method was applied to estimate the between-study variance (τ^2^). I^2^ values of 25%, 50%, and 75% were considered indicative of small, moderate, and high levels of heterogeneity, respectively [14].

### 2.6. Study Endpoints

The primary endpoint was the occurrence of net adverse clinical event (NACE), a composite of all-cause death, major vascular complications, and major bleeding at 30-day follow-up.

Secondary endpoints included all-cause death, cardiovascular death, major vascular complications, major bleeding, any bleeding, stroke, non-fatal myocardial infarction (MI), and the need for red packed blood cells at 30-day follow-up. A qualitative synthesis of the results is provided in Table 1.

## 3. Results

### 3.1. Qualitative Analysis

A total of nine full-text articles were assessed for eligibility. Of these, six studies were excluded for the following reasons: two were protocols with no results, two did not include an indication for OACs, and two did not report relevant outcomes of interest.

**Figure 1:**
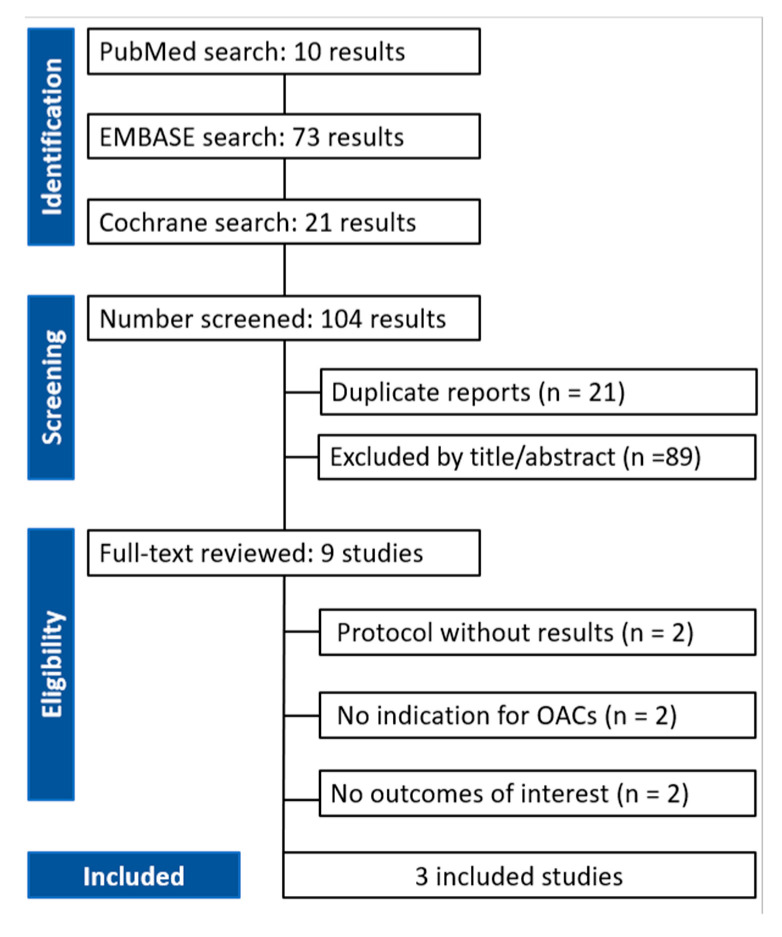
PRISMA flow diagram of study screening and selection.

Ultimately, three studies met the inclusion criteria and were included in the final analysis. The flowchart of the systematic search is illustrated in Figure 1. The most relevant clinical and procedural findings from the included articles are summarized in Table 1.

A total of three studies met the inclusion criteria and were included in the qualitative and quantitative analysis. Of these, one was a randomized clinical trial, and two were observational studies. The present systematic review included results from 2773 patients who received clinically indicated OAC therapy and underwent successful TAVI: 1314 were allocated to continuation of OAC therapy and 1459 to interruption of OAC therapy during TAVI.

AF was the primary indication for OAC therapy in all the included studies (ranging from 95% to 100%). Among the included studies, only one—the POPular TAVI trial—reported the type of atrial fibrillation at baseline. In this study, paroxysmal atrial fibrillation was present in 192 of 414 patients (46.4%) in the continuation group and 184 of 406 patients (45.3%) in the interruption group. In the continuation group, direct oral anticoagulants (DOACs) were the most commonly used anticoagulants in two studies, while in the study by Brinkert et al. DOACs and VKAs were equally represented.

The prevalence of hypertension and diabetes varied from 77% to 97% and 29% to 48%, respectively, while a history of stroke was observed in 13% to 22% of the included populations.

The results of the quantitative analysis are graphically displayed using Forest plots (Figure 2, Figure 3, Figure 4, Figure 5, Figure 6, Figure 7, Figure 8, Figure 9 and Figure 10).

### 3.2. Risk of Bias Assessment

The major findings of the risk-of-bias assessment are reported in Supplementary Table S1. One open-label study was considered at low risk of bias, and two had some concerns.

### 3.3. Quantitative Analysis

Nine meta-analyses were performed to compare the safety and efficacy of continuation versus interruption of OAC therapy during TAVI. Three studies reported data on NACE, all-cause death, major vascular complications, major bleeding, any bleeding, and stroke, while two investigated the occurrence of cardiovascular death, non-fatal MI, and the need for red packed blood cells.

**Figure 2:**
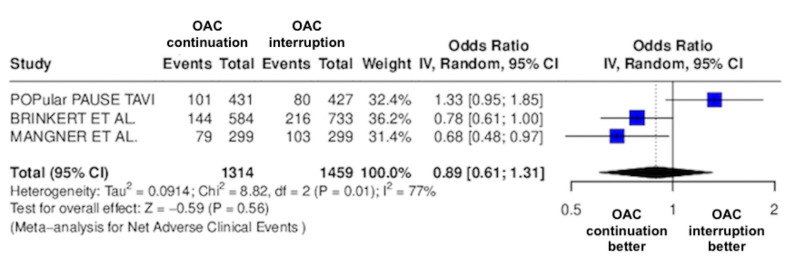
Odds ratio of NACE between the continuation OAC group and the interruption OAC group [10,11,12].

**Figure 3:**
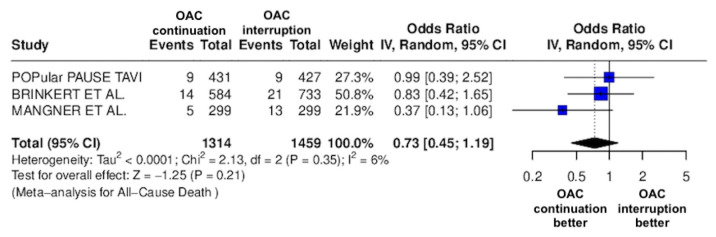
Odds ratio of all-cause death between the continuation OAC group and the interruption OAC group [10,11,12].

**Figure 4:**
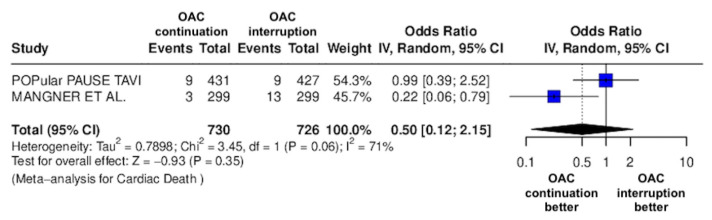
Odds ratio of cardiac death between the continuation OAC group and the interruption OAC group [11,12].

**Figure 5:**
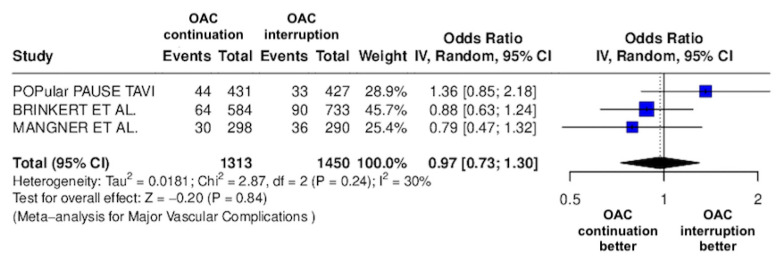
Odds ratio of major vascular complications between the continuation OAC group and the interruption OAC group [10,11,12].

**Figure 6:**
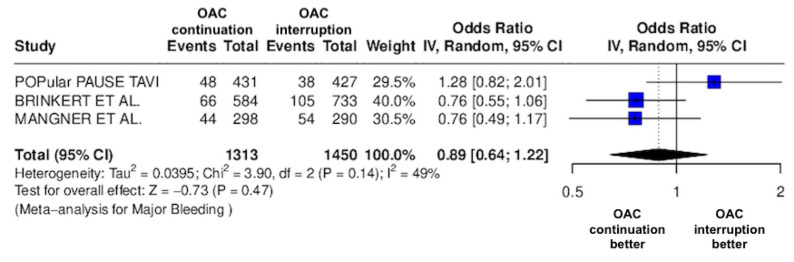
Odds ratio of major bleeding between the continuation OAC group and the interruption OAC group [10,11,12].

**Figure 7:**
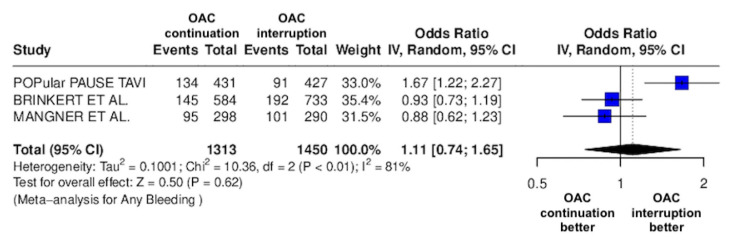
Odds ratio of total bleeding events between the continuation OAC group and the interruption OAC group [10,11,12].

**Figure 8:**
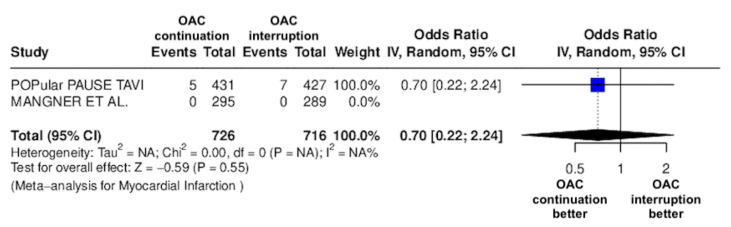
Odds ratio of non-fatal MI between the continuation OAC group and the interruption OAC group [11,12].

**Figure 9:**
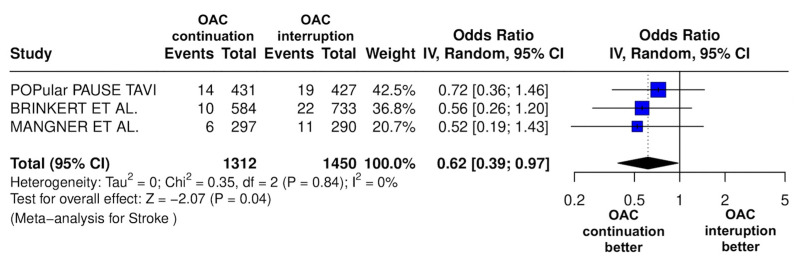
Odds ratio of stroke between the continuation OAC group and the interruption OAC group [10,11,12].

**Figure 10:**
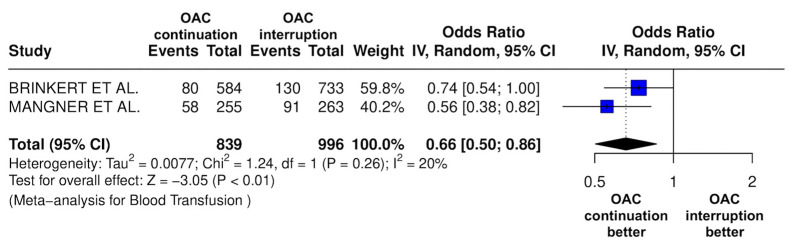
Odds ratio of the need for red packed blood cells between the continuation OAC group and the interruption OAC group [10,12].

The continuation OAC group experienced a similar rate of NACE (OR = 0.89 [95% CI 0.61 to 1.31], I^2^ = 77%, *p* = 0.56, Figure 2) as the interruption OAC group. There were no significant differences between the continuation OAC group and the interruption OAC group for the occurrence of all-cause death (OR = 0.73 [95% CI 0.45 to 1.19], I^2^ = 6%, *p* = 0.21, Figure 3), cardiovascular death (OR = 0.50 [95% CI 0.12 to 2.15], I^2^ = 71%, *p* = 0.35, Figure 4), major vascular complications (OR = 0.97 [95% CI 0.73 to 1.30], I^2^ = 30%, *p* = 0.84, Figure 5), major bleeding events (OR = 0.89 [95% CI 0.64 to 1.22], I^2^ = 49%, *p* = 0.47, Figure 6), any bleeding events (OR = 1.11 [95% CI 0.74 to 1.65], I^2^ = 81%, *p* = 0.62, Figure 7), or non-fatal MI (OR = 0.70 [95% CI 0.22 to 2.24], *p* = 0.55, Figure 8). Interestingly, the continuation OAC group experienced a lower occurrence of stroke (OR = 0.62 [95% CI 0.39 to 0.97], I^2^ = 0%, *p* = 0.04, Figure 9) and the need for red packed blood cells (OR = 0.66 [95% CI 0.50 to 0.86], I^2^ = 20%, *p* < 0.01, Figure 10) compared to the interruption OAC group (Table 2).

## 4. Discussion

To our knowledge, this is the first systematic review and meta-analysis comparing the efficacy and safety of continuation versus interruption of OACs during the periprocedural phase of TAVI in patients with a concomitant clinical indication for OAC.

The main findings from the pooled analyses were as follows: (1) there was no significant difference between continuation and interruption of OACs in terms of NACE, all cause-death, cardiovascular death, major vascular complications, major bleeding events, any bleeding events, or non-fatal MI; and (2) the continuation OAC group showed a lower occurrence of stroke and a lower need of red packed blood cells compared to the continuation group.

Despite advancements in technical skills and the minimization of delivery system caliber, the risk of adverse events during the periprocedural phase of TAVI remains significant. Patients are often frail, particularly those receiving OACs, with a 30-day adverse event rate of 15.6%, as reported in the study by Van Ginkel et al. [11].

Until a few years ago, interruption of OAC therapy was considered the best option to minimize the risk of major periprocedural bleeding due to the use of large devices for vascular access, especially in frail patients. However, emerging evidence has challenged this approach, and current guidelines remain unclear regarding the optimal management of OAC therapy during the periprocedural phase of TAVI. Therefore, real-world clinical practice involves a variety of approaches, with different OAC types (DOACs vs. VKAs) being used, and the decision to continue or interrupt therapy largely remains at the discretion of the operator.

The results of our meta-analysis suggest that continuing OAC therapy is as safe and effective as interrupting it. The rate of NACE at 30 days was comparable between the two groups (24.7% vs. 27.3% in the OAC continuation group vs. 27.3%, 399/1459 in the OAC interruption group). However, high heterogeneity across studies may influence the reliability of these results.

It is worth noting that several factors related to patient characteristics and procedural features contribute to the occurrence of adverse events [5,6]. A meticulous TAVI planning strategy aimed at minimizing vascular access and device sizes may have prognostic relevance [15].

A major finding of our meta-analysis is that continuing OAC therapy during the periprocedural phase of TAVI was associated with a lower incidence of early stroke events. This is not surprising, as atrial fibrillation is an independent predictor of stroke after TAVI [16], and aggressive OAC therapy may have prognostic relevance in this population.

Of note, our results highlighted that the OAC continuation group required fewer red blood cell transfusions compared to the OAC interruption group. Although mechanistic explanations cannot be definitively drawn from our results, the lack of bridging therapy with heparin may help explain these findings. Previous studies have shown that bridging therapy with heparin is associated with higher rates of pocket hematomas in patients undergoing pacemaker or defibrillator surgery (Essebag et al., 2019) [16] as well as increased minor bleeding in patients undergoing catheter ablation of AF [17] compared to the OAC continuation strategy.

One possible contributor to the high heterogeneity observed in bleeding outcomes (I^2^ = 81%) is the variability in the type of oral anticoagulation used across studies. For instance, Mangner et al. provided detailed breakdowns of both VKAs and various DOACs (rivaroxaban, apixaban, edoxaban, dabigatran), whereas the POPular TAVI trial did not specify the anticoagulant type used. Brinkert et al. included patients on both VKAs and DOACs but did not distinguish outcomes based on anticoagulant class. Given the known differences in bleeding risk profiles between VKAs and DOACs—and even among individual DOACs—this variability likely influenced the pooled bleeding estimates and contributed to the observed heterogeneity. Another factor likely contributing to the high heterogeneity in bleeding outcomes (I^2^ = 81%) is the use of different bleeding classification systems across studies. While Brinkert et al. and Magnéner et al. utilized the VARC criteria, the POPular TAVI trial used the BARC definition. These bleeding scales differ in thresholds and categorization of events, which may have led to inconsistencies in event reporting and outcome interpretation across the studies.

The observed heterogeneity in NACE outcomes (I^2^ = 77%) may also stem from differences in how this composite endpoint was defined across studies. In Brinkert et al., NACE included vascular complications, stroke, and mortality. The POPular TAVI trial used a broader composite that included cardiovascular death, stroke, myocardial infarction, major vascular complications, and major bleeding. Meanwhile, Magnéner et al. defined NACE as a combination of life-threatening or major bleeding, stroke, and all-cause mortality. These inconsistencies in endpoint definitions can significantly influence event rates and compromise direct comparability across studies.

### Limitations

This study has several limitations. First, the analysis included only three randomized and observational studies with a relatively low number of patients. Second, OAC therapy included both DOACs and VKAs, which differ significantly in their pharmacokinetic properties, potentially introducing bias. Third, several factors contributing to adverse outcomes, such as the use of embolic protection devices, the type of vascular closure devices, and the percentage of patients receiving bridging therapy with heparin, were not accounted for in the analysis. Finally, the lack of patient-level data precluded the identification of individuals who might derive greater benefit from continuing OAC therapy during the periprocedural phase of TAVI.

One important limitation is that the type of atrial fibrillation—whether paroxysmal, persistent, or permanent—was not specified in the included studies, with the exception of the POPular TAVI trial. This limits our ability to evaluate the potential impact of AF subtype on clinical outcomes. Given that different AF types are associated with varying risks of thromboembolism and bleeding, the distribution of AF subtypes may influence both procedural and long-term outcomes, as well as the risk–benefit balance of continuing versus interrupting anticoagulation.

Another limitation is the lack of data regarding the presence of bicuspid aortic valves across the included studies. None of the trials reported whether patients had bicuspid morphology at baseline, which is known to be associated with altered hemodynamics and a potentially higher risk of valve thrombosis. This limits our ability to assess whether bicuspid anatomy may have influenced outcomes related to anticoagulation continuation or interruption.

Information regarding the specific type of transcatheter heart valve (THV) implanted was not consistently reported across the included studies. While Brinkert et al. and Mangner et al. stated that CE-marked valves were used, they did not specify whether balloon-expandable or self-expanding valves were employed. The POPular TAVI trial did not report valve type at all. Given that valve type can influence procedural complexity, bleeding risk, and post-procedural outcomes, this lack of detail may have contributed to residual confounding and limited our ability to explore valve-related outcome differences. Furthermore, there are no data about the dimensions of the valves implanted in the patients.

## 5. Conclusions

No statistical significance between the continuation and interruption of OACs in terms of NACE was observed in the present meta-analysis. These results are adequate for any adult age group, gender, or indication for OACs. However, given that only three studies were included, the statistical power and generalizability of the findings are limited. Therefore, the results cannot be generalized across all age groups, genders, or indications for OAC use. Future randomized studies focusing on DOACs with different dosages are required to identify the optimal periprocedural management of OAC therapy in patients with AF scheduled for TAVI.

## Figures and Tables

**Table 1:** Characteristics of included studies.

Author, Year	Source Population	Primary Objective	Exclusion Criteria	Primary Outcomes	Duration of Follow-Up	Main Results
POPular PAUSE TAVI [11]	22 European sites	A non-inferiority RCT to explore the short-term outcomes of continuing vs. interrupting oral anti-coagulation in patients undergoing TAVI.	Mechanical heart valve prosthesis, intracardiac thrombus, venous thromboembolism within 3 months prior to TAVI, or transient ischemic attack or stroke in patients with atrial fibrillationwithin 6 months prior to TAVI	Composite of death from cardiovascular causes, stroke from any cause, myocardial infarction, major vascular complications, or major bleeding within 30 days after TAVI	30 days	Non-inferiority is not met concerning primary outcomes. Certain groups in the subanalysis (high CHA₂DS₂-VASc and previous stroke) showed a slight effect on the primary endpoints, favoring the continuation of anticoagulation, but it remains a hypothesis-generating observation.
Brinkert et al. [10]	5 high volume European centers (Heart Center Lucerne, Switzerland; Heart Center Leipzig at University of Leipzig, Germany; University Hospital Bern, Switzerland; University Hospital Zurich, Switzerland; and University Central Hospital Helsinki, Finland)	A retrospective study comparing periprocedural continuation of oral anticoagulation to interrupted anticoagulation in terms of safety and efficacy.	N/A	Major life-threatening bleeding at 30 days	30 days and 1 year	Major life-threatening bleeding was similar between the continuation and interruption anticoagulation groups. PRBC is less needed in the continuation of the anticoagulation group. The stroke rate difference is statistically nonsignificant between the two groups, with a numerical trend favoring the continuation of the anticoagulation group.
Mangner et al. [12]	Single-center study	Retrospective non-randomized study comparing 3 anticoagulation strategies: interrupted VKA, VKA, and DOAC	N/A	Early safety at 30 days (composite of all-cause mortality, all stroke, life-threatening bleeding, acute kidney injury stage 2 and 3, coronary obstruction requiring intervention, major vascular complication, and valve-related dysfunction requiring repeat procedure)	30 days and 1 year	Continued VKA did not increase the rate of primary endpoints at 30 days. DOAC showed a decreased rate of early safety events and improved mortality rate at 1 year.

**Table 2:** Summary of the Baseline Characteristics.

Variable	POPular PAUSE TAVI		Brinkert et al.		Mangner et al.	
	Continuation of Anticoagulation	Interrupted Anticoagulation	Continuation of Anticoagulation (*n* = 584)	Interrupted Anticoagulation (*n* = 733)	Continuation of Anticoagulation (*n* = 299) ****	Interrupted Anticoagulation (*n* = 299)
Age	81.4 ± 5.6	80.9 ± 6.2	82 (78–85)	82 (78–86)	80	80 (76–83)
Female sex (%)	158 (36.7)	138 (32.3)	287 (49)	382 (52)	164	172 (57.5)
MBI	26.5 (24.2–29.7)	26.9 (24.3–30.8)	27.1 (24–30.8) (*n* = 580)	27.1 (24–30.8) (*n* = 731)	27.75	27.9 (25–32)
EUROSCORE II	3.8 ± 3.9	3.9 ± 4.3	N/A	N/A	N/A	N/A
STS-PROM	N/A	N/A	4.8 (3.2–7.8) (*n* = 580)	5.0 (3.3–7.7) (*n* = 709)	6.3 **	6.9 (4.2–10.8) **
NYHA 1 (%)	11 (2.6)	15 (3.5)	N/A	N/A	N/A	N/A
NYHA 2 (%)	152 (35.3)	146 (34.2)	N/A	N/A	N/A	N/A
NYHA 3 (%)	241 (55.9)	238 (55.7)	N/A	N/A	216 (*n*= 298)	238 (81.2) (*n*= 293)
NYHA 4 (%)	27 (6.3)	28 (6.6)	N/A	N/A		
Atrial fibrillation	414 (96.1)	406 (95.1)	500 (97)	644 (94)	299	299 (100)
CHA2DS2-VASc score	4.5 ± 1.4	4.4 ± 1.4	5 (4–6) (*n* = 537)	5 (4–6) (*n* = 682)	5	6 (5–6)
Hypertension	339 (78.7)	322 (75.4)	525 (90)	664 (91)	291	289 (96.7)
Diabetes	128 (29.7)	123 (28.8)	204 (35) (*n* = 583)	277 (38) (*n* = 733)	126	162 (54.2)
Coronary artery disease	207 (48)	206 (48.2)	N/A	N/A	126 (*n* = 298)	131 (43.8)
History of MI	61 (14.2)	75 (17.6)	N/A	N/A	27	46 (15.4)
Previous stroke	88 (20.3) *	101 (23.6)	90 (15)	118 (16)	48	39 (13)
Peripheral artery disease	79 (18.3)	85 (19.9)	75 (13)	88 (12)	41	33 (11)
COPD	68 (15.8)	49 (11.5)	N/A	N/A	47	47 (15.7)
CKD	213 (49.4)	221 (51.8)	N/A	N/A	95 (*n* = 297)	101 (33.9) *** (*n* = 298)
Previous AVR	36 (8.4)	28 (6.6)	N/A	N/A	18	10 (3.3)
Previous PM implantation	75 (17.4)	88 (20.6)	114 (20)	101 (14)	N/A	N/A
Cerebral embolic protection device	47	38	19	17	N/A	N/A
Self-expanding	N/A	N/A	N/A	N/A	192	212 (70.9)
Balloon expending	N/A	N/A	N/A	N/A	107	87 (29.1)

* TIA, ischemic stroke, hemorrhagic stroke, and unknown origin. ** STS score. *** CKD ≥ 3B. N/A: not applicable. **** In this study, there was a separate group. The first is continued VKA, and the second is DOAC. In our table, these groups are combined.

## Data Availability

All data generated or analyzed during this study are included in this published article.

## Supplementary Materials

The following supporting information can be downloaded at: https://www.mdpi.com/article/10.3390/jcm14103563/s1, Table S1: Risk of bias summary for randomized studies (RoB 2).

## Abbreviations

The following abbreviations are used in this manuscript:

AF	Atrial Fibrillation
OAC	Oral Anticoagulation
TAVI	Transcatheter Aortic Valve Implantation
NACE	Net Adverse Clinical Events
AKI	Acute Kidney Injury
ESRD	End-Stage Renal Disease
PCI	Percutaneous Coronary Intervention
VKA	Vitamin K Antagonist
DOAC	Direct Oral Anticoagulant
MI	Myocardial Infarction
MACE	Major Adverse Cardiovascular Events
ICH	Intracranial Hemorrhage
RCT	Randomized Controlled Trial
HR	Hazard Ratio
CI	Confidence Interval
INR	International Normalized Ratio
TIA	Transient Ischemic Attack
PVL	Paravalvular Leak
RoB	Risk of bias
