# Supplementary material for: Peri-Procedural Continuation Versus Interruption of Anticoagulation for Transcatheter Aortic Valve Implantation: A Systematic Review and Meta-Analysis
Source: J Clin Med. 2025 May 20;14(10):3563. doi: 10.3390/jcm14103563 (PMC12112029; doi:10.3390/jcm14103563)
Supplement: Supplementary file 1 [file jcm-14-03563-s001.zip › jcm-3569349-supplementary.pdf]

Supplementary Material:

Supplementary Table 1: Risk of bias summary for randomized studies (RoB 2)

Study	Bias from the randomization process	Bias due to deviations from intended interventions	Bias due to missing outcome data	Bias in the measurement of the outcomes	Bias in the selection of the reported result	Overall risk of bias
PoPOPular PAUSE TAVI	Low	Low	Low	Low	Low	Low
BRINKERT ET AL	Some concerns	Low	Low	Low	Low	Some concerns
MANGNER ET AL	Some concerns	Low	Low	Low	Low	Some concerns
